# Supplementary material for: Endobronchial ultrasound-guided transbronchial needle aspiration versus mediastinoscopy for mediastinal staging of lung cancer: A systematic review of economic evaluation studies
Source: PLoS One. 2020 Jun 30;15(6):e0235479. doi: 10.1371/journal.pone.0235479 (PMC7326228; doi:10.1371/journal.pone.0235479)
Supplement: S1 File — Search Keys used based on the PICO strategy. (PDF) [file pone.0235479.s002.pdf]

PICO	
**Pacient / Problem**	"Lung Neoplasms" OR "Pulmonary Neoplasms" OR "Neoplasms, Lung" OR "Lung Neoplasm" OR "Neoplasm, Lung" OR "Neoplasms, Pulmonary" OR "Neoplasm, Pulmonary" OR "Pulmonary Neoplasm" OR "Lung Cancer" OR "Cancer, Lung" OR "Cancers, Lung" OR "Lung Cancers" OR "Pulmonary Cancer" OR "Cancer, Pulmonary" OR "Cancers, Pulmonary" OR "Pulmonary Cancers" OR "Cancer of the Lung" OR "Cancer of Lung"<br><br>OR<br><br>"Staging, Neoplasm" OR "Tumor Staging" OR "Staging, Tumor" OR "Cancer Staging" OR "Staging, Cancer" OR "TNM Staging" OR "Staging, TNM" OR "TNM Staging System" OR "Staging System, TNM" OR "Staging Systems, TNM" OR "System, TNM Staging" OR "Systems, TNM Staging" OR "TNM Staging Systems" OR "TNM Classification" OR "Classification, TNM" OR "Classifications, TNM" OR "TNM Classifications"
**Intervention**	EBUS-TBNA OR EBUS OR "endobronchial ultrasound" OR “endobronchial ultrasonography” OR "endobronchial ultrasound-guided" OR "transbronchial needle aspiration" OR "endobronchial endosonography" OR "minimally invasive endoscopic staging" OR "minimally invasive methods for mediastinal staging"
**Comparator**	Mediastinoscopy OR Mediastinoscopies OR "Mediastinoscopic Surgical Procedures" OR "Mediastinoscopic Surgical Procedure" OR "Procedure, Mediastinoscopic Surgical" OR "Procedures, Mediastinoscopic Surgical" OR "Surgical Procedure, Mediastinoscopic" OR "Surgery, Mediastinoscopic" OR "Surgical Procedures, Mediastinoscopic" OR "Mediastinoscopic Surgery" OR "Mediastinoscopic Surgeries" OR "Surgeries, Mediastinoscopic"
**Outcome**	Economics[Mesh:NoExp] OR "Costs and Cost Analysis"[mh] OR Economics, Nursing[mh] OR Economics, Medical[mh] OR Economics, Pharmaceutical[mh] OR Economics, Hospital[mh] OR Economics, Dental[mh] OR "Fees and Charges"[mh] OR Budgets[mh] OR budget*[tiab] OR economic*[tiab] OR cost[tiab] OR costs[tiab] OR costly[tiab] OR costing[tiab] OR price[tiab] OR prices[tiab] OR pricing[tiab] OR pharmacoeconomic*[tiab] OR pharmaco-economic*[tiab] OR expenditure[tiab] OR expenditures[tiab] OR expense[tiab] OR expenses[tiab] OR financial[tiab] OR finance[tiab] OR finances[tiab] OR financed[tiab] OR value for money[tiab] OR monetary value*[tiab] OR models, economic[mh] OR economic model*[tiab] OR markov chains[mh] OR markov[tiab] OR monte carlo method[mh] OR monte carlo[tiab] OR Decision Theory[mh] OR decision tree*[tiab] OR decision analy*[tiab] OR decision model*[tiab]
