# Supplementary material for: Endobronchial ultrasound-guided transbronchial needle aspiration versus mediastinoscopy for mediastinal staging of lung cancer: A systematic review of economic evaluation studies
Source: PLoS One. 2020 Jun 30;15(6):e0235479. doi: 10.1371/journal.pone.0235479 (PMC7326228; doi:10.1371/journal.pone.0235479)
Supplement: S2 File — Search strategy used on Medline (Pubmed). (PDF) [file pone.0235479.s003.pdf]

Search Strategy		
**Database**	**Strategy**	**Results**
PUBMED	(((("Lung Neoplasms"[Title/Abstract] OR "Pulmonary Neoplasms"[Title/Abstract] OR "Lung Neoplasm"[Title/Abstract] OR "Neoplasm, Lung"[Title/Abstract] OR "Pulmonary Neoplasm"[Title/Abstract] OR "Lung Cancer"[Title/Abstract] OR "Cancer, Lung"[Title/Abstract] OR "Lung Cancers"[Title/Abstract] OR "Pulmonary Cancer"[Title/Abstract] OR "Pulmonary Cancers"[Title/Abstract] OR "Cancer of the Lung"[Title/Abstract] OR "Cancer of Lung"[Title/Abstract] OR "Tumor Staging"[Title/Abstract] OR "Cancer Staging"[Title/Abstract] OR "TNM Staging"[Title/Abstract] OR "TNM Staging System"[Title/Abstract] OR "TNM Staging Systems"[Title/Abstract] OR "TNM Classification"[Title/Abstract] OR "Classification, TNM"[Title/Abstract] OR "TNM Classifications"[Title/Abstract]) OR "Lung Neoplasms"[MeSH Terms]) AND (EBUS-TBNA[Title/Abstract] OR EBUS[Title/Abstract] OR "endobronchial ultrasound"[Title/Abstract] OR "endoscopic ultrasound"[Title/Abstract] OR "endobronchial ultrasonography"[Title/Abstract] OR "endosonography"[Title/Abstract] OR "endobronchial ultrasound-guided"[Title/Abstract] OR "transbronchial needle aspiration"[Title/Abstract] OR "fine needle aspiration"[Title/Abstract] OR "minimally invasive endoscopic staging"[Title/Abstract])) AND (("mediastinoscopy"[MeSH Terms] OR "mediastinoscopy"[All Fields]) OR ("mediastinoscopy"[MeSH Terms] OR "mediastinoscopy"[All Fields] OR "mediastinoscopies"[All Fields]) OR ("mediastinoscopy"[MeSH Terms] OR "mediastinoscopy"[All Fields] OR ("mediastinoscopic"[All Fields] AND "surgical"[All Fields] AND "procedures"[All Fields])) OR ("mediastinoscopy"[MeSH Terms] OR "mediastinoscopy"[All Fields] OR ("mediastinoscopic"[All Fields] AND "surgical"[All Fields] AND "procedure"[All Fields])) OR ("mediastinoscopy"[MeSH Terms] OR "mediastinoscopy"[All Fields] OR ("procedure"[All Fields] AND "mediastinoscopic"[All Fields] AND "surgical"[All Fields])) OR ("mediastinoscopy"[MeSH Terms] OR "mediastinoscopy"[All Fields] OR ("procedures"[All Fields] AND "mediastinoscopic"[All Fields] AND "surgical"[All Fields])) OR ("mediastinoscopy"[MeSH Terms] OR "mediastinoscopy"[All Fields] OR ("surgical"[All Fields] AND "procedure"[All Fields] AND "mediastinoscopic"[All Fields])) OR ("mediastinoscopy"[MeSH Terms] OR "mediastinoscopy"[All Fields] OR ("surgery"[All Fields] AND "mediastinoscopic"[All Fields])) OR ("mediastinoscopy"[MeSH Terms] OR "mediastinoscopy"[All Fields] OR ("surgical"[All Fields] AND "procedures"[All Fields] AND "mediastinoscopic"[All Fields])) OR "Mediastinoscopic Surgery"[All Fields] OR ("mediastinoscopy"[MeSH Terms] OR "mediastinoscopy"[All Fields] OR ("mediastinoscopic"[All Fields] AND "surgeries"[All Fields])) OR ("mediastinoscopy"[MeSH Terms] OR "mediastinoscopy"[All Fields] OR ("surgeries"[All Fields] AND "mediastinoscopic"[All Fields])))) AND ("economics"[MeSH Terms:noexp] OR "Costs and Cost Analysis"[mh] OR "economics, nursing"[MeSH Terms] OR "economics, medical"[MeSH Terms] OR "economics, pharmaceutical"[MeSH Terms] OR "economics, hospital"[MeSH Terms] OR "economics, dental"[MeSH Terms] OR "budgets"[MeSH Terms] OR (budget[tiab] OR budget'[tiab] OR budget's[tiab] OR budgetable[tiab] OR budgetaire[tiab] OR budgetarily[tiab] OR budgetary[tiab] OR budgeted[tiab] OR budgeteering[tiab] OR budgeters[tiab] OR budgetfor[tiab] OR budgetierung[tiab] OR budgeting[tiab] OR budgeting'[tiab] OR budgeting's[tiab] OR budgetizing[tiab] OR budgetrestricted[tiab] OR budgetry[tiab] OR budgets[tiab] OR budgets'[tiab] OR budgetsis[tiab] OR budgett[tiab] OR budgett's[tiab] OR budgetting[tiab]) OR (economic[tiab] OR economic'[tiab] OR economic''[tiab] OR economic's[tiab] OR economica[tiab] OR economical[tiab] OR economical'[tiab] OR economicall[tiab] OR economically[tiab] OR economically'[tiab] OR economicallyoriented[tiab] OR economicallyuncovered[tiab] OR economicalness[tiab] OR economicaly[tiab] OR economicamente[tiab] OR economicas[tiab] OR economicbranches[tiab] OR economiche[tiab] OR economici[tiab] OR economicissue[tiab] OR economicist[tiab] OR economicist'[tiab] OR economicity[tiab] OR economiclly[tiab] OR economicmicro[tiab] OR economico[tiab] OR economicomathematical[tiab] OR economicos[tiab] OR economicosocial[tiab] OR economicperformance[tiab] OR economicpubguidelines[tiab] OR economics[tiab] OR economics'[tiab] OR economicsanalysis[tiab] OR economicscience[tiab] OR economicus[tiab] OR economicus'[tiab] OR economicus'rationalism[tiab] OR economicwise[tiab]) OR cost[tiab] OR costs[tiab] OR costly[tiab] OR costing[tiab] OR price[tiab] OR prices[tiab] OR pricing[tiab] OR (pharmacoeconomic[tiab] OR	69

	pharmacoeconomical[tiab] OR pharmacoeconomically[tiab] OR pharmacoeconomics[tiab]) OR (pharmaco economic[tiab] OR pharmaco economical[tiab] OR pharmaco economically[tiab] OR pharmaco economics[tiab]) OR expenditure[tiab] OR expenditures[tiab] OR expense[tiab] OR expenses[tiab] OR financial[tiab] OR finance[tiab] OR finances[tiab] OR financed[tiab] OR value for money[tiab] OR (monetary value[tiab] OR monetary values[tiab]) OR "models, economic"[MeSH Terms] OR (economic model[tiab] OR economic modeling[tiab] OR economic modelling[tiab] OR economic models[tiab]) OR "markov chains"[MeSH Terms] OR markov[tiab] OR "monte carlo method"[MeSH Terms] OR monte carlo[tiab] OR "decision theory"[MeSH Terms] OR (decision tree[tiab] OR decision treeboost[tiab] OR decision trees[tiab]) OR (decision analyses[tiab] OR decision analysis[tiab] OR decision analyst[tiab] OR decision analysts[tiab] OR decision analytic[tiab] OR decision analytical[tiab] OR decision analytics[tiab]) OR (decision model[tiab] OR decision modelers[tiab] OR decision modeling[tiab] OR decision modelling[tiab] OR decision models[tiab]))	
