# Supplementary material for: Endobronchial ultrasound-guided transbronchial needle aspiration versus mediastinoscopy for mediastinal staging of lung cancer: A systematic review of economic evaluation studies
Source: PLoS One. 2020 Jun 30;15(6):e0235479. doi: 10.1371/journal.pone.0235479 (PMC7326228; doi:10.1371/journal.pone.0235479)
Supplement: S3 File — Publications evaluated according to the CHEERS checkpoint. (PDF) [file pone.0235479.s004.pdf]

	Title Identified as economic evaluation	Structured abstract	Introduction provides context and a clear study question	Population characteristics	Setting and location	Study Perspective	Comparators described	Time horizon	Discount rate	Outcomes and relevance	Measurement of effectiveness	Preference based outcomes
**AUTHOR**	**1**	**2**	**3**	**4**	**5**	**6**	**7**	**8**	**9**	**10**	**11**	**12**
Ang et al	√	√	√	≠	≠	√	√	X	X	√	≠	N.A.
Kazia Czarnecka-Kujawa et al	√	√	√	√	√	√	√	√	X	√	√	N.A.
Harewood et al	√	√	√	√	≠	≠	√	X	X	√	≠	N.A.
Luque et al	X	√	√	√	X	√	≠	X	X	√	≠	N.A.
Navani et al	X	√	√	√	√	√	√	X	X	√	√	N.A.
Sharples et al	√	v	√	√	√	√	√	√	X	√	√	N.A.
Søgaard et al	√	√	√	√	≠	√	√	√	√	√	√	N.A.
Steinfort et al	√	√	√	√	√	≠	√	X	√	√	√	N.A.

	Resources and costs	Currency, date and conversion	Model choice described	Model assumptions	Analytical methods	Parameters of values	Incremental cost	Sensitivity of incremental costs or model sensitivity analyses	Heterogenity explained	Findings and limitations	Funding source	Potential conflict of interest
**AUTHOR**	**13**	**14**	**15**	**16**	**17**	**18**	**19**	**20**	**21**	**22**	**23**	**24**
Ang et al	≠	X	≠	√	X	√	≠	√	N.A.	≠	X	X
Kazia Czarnecka -Kujawa et al	√	√	√	√	≠	√	√	√	N.A.	√	X	X
Harewood et al	√	≠	√	√	≠	√	√	√	N.A.	√	X	√
Luque et al	≠	X	√	≠	X	√	X	√	N.A.	√	√	√
Navani et al	√	√	√	≠	≠	√	√	X	N.A.	√	X	X
Sharples et al	√	√	N.A.	N.A.	√	√	√	√	N.A.	√	√	X
Søgaard et al	√	√	√	√	≠	√	√	√	N.A.	√	√	X
Steinfort et al	√	√	√	√	≠	√	√	√	N.A.	√	√	X
