# Supplementary material for: Endobronchial ultrasound-guided transbronchial needle aspiration versus mediastinoscopy for mediastinal staging of lung cancer: A systematic review of economic evaluation studies
Source: PLoS One. 2020 Jun 30;15(6):e0235479. doi: 10.1371/journal.pone.0235479 (PMC7326228; doi:10.1371/journal.pone.0235479)
Supplement: S1 Data — Data abstraction form used for data abstraction. (DOCX) [file pone.0235479.s005.docx]

**DATA ABSTRACTION FORM**

- **Systematic review of economic evaluation studies EBUS x mediastinoscopy for mediastinal staging of lung cancer**

| - **Identification** | Reference Number |  |
| --- | --- | --- |
| - **Identification** | Title |  |
| - **Identification** | Author |  |
| - **Identification** | Year |  |
| - **Identification** | Journal Name |  |
| - **Identification** | Country |  |
| - **Type of Study** | Type of Study |  |
| - **Study Design** | Study Design |  |
| - **Population** | Characteristics |  |
| - **Population** | Number of patients |  |
| - **Population** | Average age |  |
| - **Population** | Median age |  |
| - **Study Perspective** | Study perspective |  |
| - **Time Horizon** | Time horizon |  |
| - **Intervention** | Intervention |  |
| - **Comparators** | Comparators |  |
| - **Parameters of the staging procedure** | Measure of effectiveness |  |
| - **Data source** | Data source |  |
| - **Costs** | Cost types |  |
| - **Costs** | Items included in costs |  |
| - **Costs** | Source of cost data |  |
| - **Costs** | Year accounted |  |
| - **Costs** - **Costs** | Inflation rate  Currency unit |  |
| - **Costs** | Total costs of intervention |  |
| - **Costs** | Total costs of comparator |  |
| - **Costs** | Cost Reduction |  |
| - **Model** | Model used |  |
| - **Model** | Model software |  |
| - **Model** | Parameters used in the model |  |
| - **Model** | Model data source |  |
| - **Model** | Assumptions used in the model |  |
| - **Model** | Model validation |  |
| - **Discounts** | Discounts application |  |
| - **Discounts** | Discount rate |  |
| - **Outcomes** | Outcomes |  |
| - **Sensitivity analysis** | Type of Sensitivity Analysis |  |
| - **Sensitivity analysis** | Variables tested |  |
| - **Sensitivity analysis** | Results |  |
| - **Threshold of cost-effectiveness in the country of origin** | Threshold of cost-effectiveness in the country of origin |  |
| - **Conclusion** | Conclusions |  |
| - **Relevant characteristics identified** | Relevant characteristics identified |  |
