# Supplementary material for: Endobronchial ultrasound-guided transbronchial needle aspiration versus mediastinoscopy for mediastinal staging of lung cancer: A systematic review of economic evaluation studies
Source: PLoS One. 2020 Jun 30;15(6):e0235479. doi: 10.1371/journal.pone.0235479 (PMC7326228; doi:10.1371/journal.pone.0235479)
Supplement: Attachment — Submitted filename: RESPONSE TO REVIEWERS .docx [file pone.0235479.s006.docx]

**RESPONSE TO REVIEWERS**

Dear academic editor and reviewers,

Thank you very much for taking the time to review the article “**Endobronchial ultrasound-guided transbronchial needle aspiration versus mediastinoscopy for mediastinal staging of lung cancer: systematic review of economic evaluation studies”** submitted to Plos One. I am sure that the comments and suggestions made will improve the scientific quality of the article and interest for future readers.

1. Responses to the points raised by the academic editor:

- **The authors should consider including a funnel plot to their results** – the following paragraph was added to the manuscript on lines 311-321: “Risk of bias across studies is particularly relevant when a systematic review combines evidence on treatment effects across multiple studies. However, our review seeks to evaluate the results, methodological and reporting quality of economic evaluation studies, rather than the effect of any particular intervention, and did not combine results across studies. Tools for assessing publication or selective reporting bias (i.e. funnel plots) have been designed for examining treatment effect of interventions, which cannot be applied to our study. We minimized publication bias by searching available protocols for economic evaluation studies of EBUS versus mediastinoscopy in systematic review registries available (ie. Prospero database). Additional assessment of the risk of bias across studies was also based on evaluations of each study’s funding source and the nature of disclosed conflict of interest for each study.”
- **The cost-effectiveness and cost-savings data should be combined in a separate table –** Table 3 was divided into 2 tables (table 3 and table 4). Table 3 presents the interventions, comparators and outcomes of each study and Table 4 shows the results of cost-effectiveness, cost reduction and sensitivity analysis. Changes were made to the text of the manuscript with the inclusion of a sub-section with the title “Intervention, comparators and outcomes” and another with the title “Cost-effectiveness, cost-savings and sensitivity analysis results. The main information in the tables is described in these 2 subsections.
- **While the authors demonstrate that EBUS is cost-effective compared to mediastinoscopy, the potential limitations of EBUS should be included in the discussion** – the potential limitation of EBUS were included in the discussion as follows on line 342 to 354: “Finally, although not addressed in the studies evaluated by this systematic review, it is important to note that the EBUS technique is highly operator dependent and the results of a service with little experience can differ greatly from those published in the literature by experts. A complete mediastinal staging approach with systematic sampling of all multiple lymph node stations is quite different and more difficult to do than just sampling one suspected lymph node. Ensuring adequate training and quality control of the results obtained by EBUS is essential for the establishment of reference centers in the technique (34). In this context, although evidence is insufficient to recommend that rapid onset evaluation (ROSE) should be used in every procedure (35), the presence of the pathologist in the bronchoscopy room can be of great value in guiding less experienced operators in obtaining representative lymph node samples. In cases where the clinical suspicion of mediastinal node involvement remains high after a negative result using a needle technique, surgical staging with mediastinoscopy should be considered (3).”
- **Please have the paper reviewed by a native English speaker. There are several typographical and grammatical errors in the manuscript as pointed out by the reviewers** – the manuscript was reviewed by a native English speaker and the typographical and grammatical errors were corrected.

2. Responses to the points raised by Reviewer 1:
   - **In regard to the data abstraction, it seems there was not structured data abstraction form used** - A structured data abstraction form was used and the form can now be accessed in the supporting information session. This information is now written in the manuscript as follows on lines 113-14: “A structured data abstraction form was used and can also be accessed in the supporting information session of this article.”
   - **Please consider including a clinically relevant and focused main study question. The objective is stated, but what is the authors main question to solve? This needs to be explicitly stated and will need to describe primary and secondary objectives** – The main study question and description of primary and secondary objectives were included as follows on lines 69 – 71 “An important question to be answered at this point is: is the use of EBUS for the mediastinal staging of lung cancer cost-effective when compared to mediastinoscopy?”and 73 – 80 “The primary objective of this study is to understand the cost-effectiveness ratio of EBUS compared to mediastinoscopy for invasive mediastinal staging of lung cancer. Secondary objectives are to identify the most relevant economic evaluation studies published comparing EBUS to mediastinoscopy and what types of models were used in those evaluations, to understand the most important economic trade-offs and to guide future economic assessments to be carried out in countries with different health systems.”
   - **Please consider adding if the cost /effectiveness of EBUS vs mediastinoscopy has NOT been clearly demonstrated in prior clinical trials** – This information was added to the manuscript on lines 73-74: “but until now the cost-effectiveness of EBUS versus mediastinoscopy has not been clearly demonstrated in prior clinical trials.”

- **Please explain if a funnel plot was used** - the following paragraph was added to the manuscript on lines 311-321: “Risk of bias across studies is particularly relevant when a systematic review combines evidence on treatment effects across multiple studies. However, our review seeks to evaluate the results, methodological and reporting quality of economic evaluation studies, rather than the effect of any particular intervention, and did not combine results across studies. Tools for assessing publication or selective reporting bias (i.e. funnel plots) have been designed for examining treatment effect of interventions, which cannot be applied to our study. We minimized publication bias by searching available protocols for economic evaluation studies of EBUS versus mediastinoscopy in systematic review registries available (ie. Prospero database). Additional assessment of the risk of bias across studies was also based on evaluations of each study’s funding source and the nature of disclosed conflict of interest for each study.”
  - **Please consider adding if the author had made attempts at collecting unpublished data** – This information was added to the manuscript on lines 99-100 as follows: “The authors chose not to include unpublished data and gray literature in the searches.”
  - **Please consider adding if a structured data abstraction form was used when selecting/scoring the studies** - A structured data abstraction form was used and the form can now be accessed in the supporting information session. This information is now written in the manuscript as follows on lines 113-14: “A structured data abstraction form was used and can also be accessed in the supporting information session of this article.”
  - **Please consider adding the size (n) of population for each study in Table 1** – The size (N) of population for each study was added in Table 1.
  - **There was repetition in the sub-section outcomes. results and sensitivity analysis’ paragraphs and Table 3. Please consider highlighting most relevant data** – The authors agreed that there was an excess of repeated information between the sub-section outcomes, results and sensitivity analysis of the manuscript and table 3. Table 3 was divided in 2 Tables (Table 3 and Table 4) and the sub-section “Outcomes, results and sensitivity analysis” was divided in 2 sub-sections: “Intervention, comparators and outcomes” and “Cost-effectiveness, cost-savings and sensitivity analysis results”. The more detailed information was taken from the text and only the messages that we consider most important from each article were left to be highlighted.
  - **Could the authors consider cost-effectiveness and cost-savings description of data be condensed on a separate table? And just include one or two shorter paragraphs to add some additional data/guide the reader?** – Table 3 was divided into 2 tables (table 3 and table 4). Table 3 presents the interventions, comparators and outcomes of each study and Table 4 shows the results of cost-effectiveness, cost-savings and sensitivity analysis. Changes were made to the text of the manuscript with the inclusion of a sub-section with the title “Intervention, comparators and outcomes” and another with the title “Cost-effectiveness, cost-savings and sensitivity analysis results”. The main information in the tables is described in these 2 subsections.
- **I would like, however ask the authors if were all these eight studies, in their opinion, combinable? If so, please add this in the discussion. I noticed that a funnel plot was not used. Was this necessary, if not, was the sensitivity analysis enough? Please explain** - the following paragraph was added to the manuscript on lines 311-321: “Risk of bias across studies is particularly relevant when a systematic review combines evidence on treatment effects across multiple studies. However, our review seeks to evaluate the results, methodological and reporting quality of economic evaluation studies, rather than the effect of any particular intervention, and did not combine results across studies. Tools for assessing publication or selective reporting bias (i.e. funnel plots) have been designed for examining treatment effect of interventions, which cannot be applied to our study. We minimized publication bias by searching available protocols for economic evaluation studies of EBUS versus mediastinoscopy in systematic review registries available (ie. Prospero database). Additional assessment of the risk of bias across studies was also based on evaluations of each study’s funding source and the nature of disclosed conflict of interest for each study.”
  - **Please consider mentioning that additionally, EUS will not be able to sample hilar lymph nodes but mediastinal** – This information was added to the manuscript on lines 337 – 340 as follows: “It is also important to note that the use of EUS has the limitation of not evaluating hilar lymph nodes, which may be important for defining the most appropriate therapeutic strategy in some cases.”
  - **Minor comments:**
  - **Abstract. Correct misspelling “statement”. Consider changing keys for keywords, pulmonary neoplasia for lung cancer or thoracic malignancy** – Correction and changes were made on lines 26 – 27 “The emergence of endobronchial ultrasound (EBUS) changed the approach to staging lung cancer” and 31 – 32 “…according to the PRISMA statement and registred on PROSPERO (**CRD42019107901)**. Search keywords were elaborated considering descriptors…” of the manuscript.
  - **Line 153, misspelling “Computer” for “Computed”. Should read Chest computed tomography** – Correction was made as follows now on lines 168-169: “…status based on chest computed tomography (CT) and positron emission tomography-computed tomography (PET-CT)…”
  - **Line 180, please spell out DRG abbreviation** – The abbreviation is now spelled as follows on line 194: “to national tariffs and/or Diagnosis Related Groups (DRG) fees (21, 23-25).”
  - **Line 209, Please clarify if the authors meant EUS or EBUS or both** – This sentence was removed from the text of the manuscript to leave only the most relevant information in the tables. Table 4 shows that: "EUS-FNA is the least expensive strategy for mediastinal lung cancer staging when N2 probability <32% / EUS + EBUS-TBNA is least expensive when N2 probability> 32%".
  - **Line 269. Maybe a typo. I only found 8 articles used, but it is written “nine”** – This is corrected on line 266 as follows: “The eight articles included in this review were…”
  - **Line 334. Delete “the”** – This is corrected on line 357 as follows: “…that EBUS-TBNA is cost effective compared to mediastinoscopy…”
  - **Table 1 - Please correct the word “cost-effectiveness”, “available”, “months”** – These corrections were made in Table 1.
  - **Consider changing “thorax CT” for “Chest CT” throughout the document** – This change was made throughout the document.
  - **Table 3 Add in authors column the corresponding # of bibliography. Consider capital letter in sensitivity analysis column. Multiple misspellings: “n.a.” and “available”, computer for “computed”** – These corrections and changes were made in Table 3.

3. Responses to the points raised by Reviewer 2:

- **Abstract intro: EBUS did not change the approach of pulmonary neoplasia. It has changed the approach to staging pulmonary neoplasia. This should be corrected throughout the manuscript** – This correction was made throughout the manuscript as follows on line 26 “The emergence of endobronchial ultrasound (EBUS) changed the approach to staging lung cancer.” and 59 “...changed the approach to staging lung cancer...”
- **There are several typos throughout the manuscript that need to be corrected prior to publication** – the manuscript was reviewed by a native English speaker and the typographical and grammatical errors were corrected.
- **I assume the column heading in Table 1 should read "Sensitivity Analysis" and not Sensitive** – The column heading in Table 1 was corrected to “Sensitivity Analysis”.
- **The discussion would be strengthened by mention of the potential limitations of EBUS, in that it is highly operator dependent, ie the time spent doing the procedure by an experienced operator systematically sampling all nodal stations will likely have significantly different outcomes in terms of nodal sampling rate, etc than one done by an inexperienced operator only sampling one node. While this is not directly related to the findings from the included studies, it could impact cost ultimately. Given that most older experienced thoracic surgeons are more familiar with mediastinoscopy than EBUS, I think the inclusion of some discussion about the importance of adequate training and quality control of EBUS, including Rapid On-site Evaluation (ROSE) of specimens should be included, even if not mentioned in the included studies** - the potential limitation of EBUS were included in the discussion as follows on line 342 to 354: “Finally, although not addressed in the studies evaluated by this systematic review, it is important to note that the EBUS technique is highly operator dependent and the results of a service with little experience can differ greatly from those published in the literature by experts. A complete mediastinal staging approach with systematic sampling of all multiple lymph node stations is quite different and more difficult to do than just sampling one suspected lymph node. Ensuring adequate training and quality control of the results obtained by EBUS is essential for the establishment of reference centers in the technique (34). In this context, although evidence is insufficient to recommend that rapid onset evaluation (ROSE) should be used in every procedure (35), the presence of the pathologist in the bronchoscopy room can be of great value in guiding less experienced operators in obtaining representative lymph node samples. In cases where the clinical suspicion of mediastinal node involvement remains high after a negative result using a needle technique, surgical staging with mediastinoscopy should be considered (3).”
